# Early childhood psychopathology and parental mental health during the COVID-19 pandemic: the effects of pandemic restrictions on 0- to 3-year-olds

**DOI:** 10.3389/frcha.2024.1441969

**Published:** 2024-11-25

**Authors:** K. Keller, S. Taubner, A. K. Georg

**Affiliations:** ^1^ Institute of Psychology, University of Heidelberg, Heidelberg, Germany; ^2^ Institute for Psychosocial Prevention, University Hospital Heidelberg, Heidelberg, Germany

**Keywords:** COVID-19 pandemic, parental mental health, regulatory disorders, child behavioral problems, early childhood psychopathology

## Abstract

**Introduction:**

The COVID-19 pandemic placed many restrictions on families and affected the mental health of parents and children. The present study examines how the restrictions imposed during the pandemic and parental mental health affect early childhood psychopathology.

**Method:**

From September 2019 to December 2021, the Outpatient Department of Family Therapy at the Institute for Psychosocial Prevention, Heidelberg surveyed a clinical sample of 249 families who sought consultation for early childhood psychopathology. Early childhood psychopathology in children aged 0–3 years was assessed using the German Questionnaire for Crying, Feeding and Sleeping and the German version of the Child Behavior Checklist 1½–5. The Patient Health Questionnaire provided information on parental depressiveness and generalized anxiety. At the same time, the Stringency Index as part of the Oxford Coronavirus Government Response Tracker indicated the severity of COVID restrictions in Germany.

**Results:**

Dependent comparisons did not reveal significant differences in the infants' regulatory problems (*n* = 165, mean age = 8 months) during the lockdown compared to reopening phases. However, older children (*n* = 84, mean age = 25 months) exhibited more behavioral problems during lockdowns compared to reopening phases (Cohen's *d* = 0.32, *p* = .04). Subsequent regression analyses confirmed a slight increase in behavioral problems only among children aged 1.5–3 years (*p* = .047, *R*^2^ = .08), but did not indicate any increase in parental mental health problems when more restrictions were in place. However, parental depressiveness had a strong independent effect on early childhood psychopathology. A hierarchical regression analysis indicated that psychopathology in children aged 1.5–3 years is best explained by female child gender, high parental depressiveness, and more severe restrictions during the COVID-19 pandemic (*p* < .001, *R*^2^ = .17) whereas early childhood psychopathology in infants aged 0-1.5 years is more prevalent in younger and male children with parents experiencing higher levels of depressiveness (*p* < .001, *R*^2^ = .26).

**Discussion:**

The study found no increase in infant regulatory disorders or parental depressiveness and generalized anxiety during the pandemic. However, older children exhibited more behavioral problems during more severe pandemic restrictions. The study supports the provision of parent-child support during crises and beyond, as early childhood psychopathology was strongly associated with parental depressiveness.

## 1 Introduction

The COVID-19 pandemic, beginning in 2020 and lasting until 2023, affected many families. To prevent the spread of the coronavirus, everyone's daily life was repeatedly restricted. Protective factors, such as leisure activities, personal social contacts, and many childcare options for young families disappeared during the resulting lockdown phases (1, 2). These constraints caused previously unprecedented stressors in many areas. Economic factors such as unemployment, along with isolation and strain, led to a spike in psychological distress among the general population (3), as well as among families and children.

Young children depend on their caregivers for help with tasks such as soothing, feeding, sleeping, and exploring, as well as during times of heightened irritation or stress (4). The authors von Hofacker et al. (5) and Charlier (6) described early childhood psychopathology in infants, namely regulatory disorders, as difficulties for young children to appropriately regulate their behavior. Early childhood psychopathology in children aged 0–3 years includes a range of symptoms such as excessive crying, sleep disturbances, feeding difficulties, defiant aggressive behavior, emotional reactivity, attention problems, depressiveness, anxiety, and social withdrawal (5, 7). The prevalence rates of specific problems vary depending on the child's age (8). For example, the prevalence of excessive crying in the first 3 months of life is approximately 16%, decreases to 6% between months 4 and 6, and is only 2.5% in 6-month-old children (8). Furthermore, gender differences have been found in aggressiveness and attention problems, with girls exhibiting fewer problems (7, 9). In infants, more boys showed regulatory problems overall (10–12), while girls appeared to cry and wake up less (13, 14).

Bronfenbrenner (15) developed a theoretical framework to explain the protective and risk factors of child development, considering environmental, situational, and interpersonal factors. The microsystem, which includes family and daycare, is the most immediate and influential system in this framework. This microsystem may have been the one most affected by the pandemic, as care options changed, and restrictions placed a considerable burden on parents. According to Tettenborn et al. (16), families self-reported childcare during the pandemic as burdensome. Bronfenbrenner's model (15) suggested that this increased burden on the parents during the pandemic may have negative effects on their children. Tettenborn et al. (16) concluded that the pandemic-related stress caused parents to lose confidence in their parenting abilities, resulting in less effective soothing attempts. The parents’ ability to co-regulate their child may have declined, increasing the risk of developing or maintaining child regulatory difficulties and behavioral problems. Difficulties in caring for children during the pandemic, such as excessive parental demands, financial difficulties, disruptions to daily routines, or conflicts within the family, could have further intensified early childhood psychopathology (4, 8, 17, 18).

Research indicates a significant increase in infant crying, longer times to fall asleep, increased sleep and crying problems, and later bedtimes during periods when COVID-related restrictions were in place (16, 19). In March 2020, during the first lockdown, infants experienced more frequent awakenings, which required parents to visit their rooms more often for comfort (20). While there is no conclusive evidence that sleep quality declined during the pandemic, the number of children not meeting a standard for adequate sleep increased (21). According to surveys conducted on feeding problems, researchers found no difference between the lockdown and reopening phases (16, 19). Feeding disorders are caused by complex somatic and psychosocial factors (22). Therefore, they may be less affected by increased family stressors in the context of the pandemic. In summary, empirical evidence suggests a correlation between the pandemic and crying and sleeping problems, whereas the evidence regarding a correlation with feeding problems is inconclusive. Furthermore, studies have shown an increase in behavioral problems in young children, such as depression, anxiety, and attention problems, with the onset of the lockdown (9, 23).

Pre-pandemic literature suggests a correlation between parental mental health and early childhood psychopathology. For instance, parental depression and related difficulties in parent-child interactions are associated with early childhood psychopathology (5, 6, 24). Empirical evidence suggests that factors such as psychosocial and prenatal stress, anxiety, postnatal depression, mental illness, substance abuse, conflict, social isolation, and family distress, such as poverty, are related to early childhood psychology (4, 8, 17, 25). Postert et al. (26) and Sidor et al. (27) confirmed the relationship between parental psychological stress and their children's regulatory disorders in two German studies. Additionally, a longitudinal study by Evers et al. (28) demonstrated reciprocal effects between early childhood psychopathology and parental stress throughout early childhood. This literature suggests that parental mental health issues and high parental stress during the pandemic may worsen early childhood psychopathology. In turn, the children's psychopathology may cause negative parental mental health responses.

Xiong et al. (3) reported a significant increase in global depression (15%–48%) and anxiety symptoms (6%–52%) during the first phase of the 2020 lockdown among adults in the general population affected by the COVID-19 pandemic. Brailovskaia and Margraf (29) identified stress as the main predictor of psychological distress during the pandemic, with anxiety and depression symptoms before the first lockdown being less predictive. A study conducted in 204 countries and territories reported a correlation between infection rates and restricted mobility with a higher incidence of anxiety and major depressive disorders (30). Women and younger adults were found to be more susceptible to anxiety and major depressive disorders than men and older individuals (30). Generalized anxiety and depressive symptoms also increased among parents (31, 32). Zhang et al. (33) discovered that postpartum mothers experienced higher levels of anxiety and depression during the pandemic. In Canada and the United States, the mental health of mothers declined even more than that of childless adults (34–36).

A study conducted between April and June 2021 found that social distancing, concerns about the child, birth anxiety, separation from the child, and exposure to COVID-19-related parenting behaviors and support, were related to increased infant psychopathology (37). This relationship was mediated by reduced maternal well-being and maternal socioemotional investment. Furthermore, empirical evidence suggests that the incidence of crying and sleep disturbances increased during the pandemic when mothers reported more depressive symptoms (19). Additionally, high levels of maternal depression and anxiety were associated with lower levels of maternal-infant bonding and infants’ regulatory ability (38, 39). Provenzi et al. (38) reported that parental stress mediated the effect of maternal postnatal anxiety on infant regulatory capacity. Similarly, quarantine in Italy was found to exacerbate behavioral and emotional problems in children aged 2–14 years, mediated by parental stress (40). Furthermore, maternal perceived stress was correlated with the time taken to calm infants, and the amount of infant crying and fussing (41).

Taken together, the pandemic has led to an increase in depression and anxiety (1, 3, 29). Previous research has also demonstrated a correlation between parental mental health and early childhood psychopathology (8, 24). It has not been conclusively examined whether lockdowns influenced early childhood psychopathology and whether this effect was mediated by parental depressiveness and generalized anxiety. Additionally, it has not been determined if these effects persist throughout early childhood. Prevalence rates depend on the age and gender of the children (7–9) which is why both were included as covariates in the following analyses. Furthermore, most studies conducted during the pandemic relied on convenient online samples. Examining a clinical sample of children with early childhood psychopathology may provide insights of direct relevance to parenting interventions in this population.

This study aims to investigate the relationship between early childhood psychopathology and COVID-19-related restrictions, considering parental mental health. The following relationships were expected.

*Hypothesis 1. Symptoms of early childhood psychopathology differ between lockdown and reopening phases.*
  *1 a. Parent-reported regulatory problems regarding crying, fussing, and sleeping are more severe during lockdown than during reopening phases.*
  *1 b. There are significant differences in parent-reported feeding difficulties between lockdown and reopening phases.*
  *1 c. Parent-reported parent-infant co-regulation difficulties regarding crying, fussing, sleeping, and feeding are more severe during lockdown than during reopening phases.*
  *1 d. Parent-reported behavioral problems are more severe during lockdown than during reopening phases.*
*Hypothesis 2. When controlling for child age and gender, pandemic restrictions predict the symptomatology of parent-reported early childhood psychopathology.*
*Hypothesis 3.* *The effect of pandemic restrictions on the symptomatology of parent-reported early childhood psychopathology is mediated in part by parents’ depressiveness and generalized anxiety symptoms.*

Finally, this study aims to exploratively identify the most relevant variables associated with early childhood psychopathology based on the data in the current study. To identify the most relevant variables, the severity of COVID-19 restrictions, parental depressiveness and generalized anxiety as well as child gender and age are included as predictors.

## 2 Methods

This cross-sectional study was conducted at the Outpatient Department of Family Therapy at the Institute for Psychosocial Prevention of the University Hospital of Heidelberg. The data collection period began on September 19, 2019, after receiving approval from the Ethics Committee of the Medical Faculty of the Heidelberg University Hospital, and before the start of the pandemic. The collection period ended on December 21, 2021.

### 2.1 Procedure

All parents of children aged 0–3 years who consulted the Outpatient Department of Family Therapy during the respective time frame were asked to fill out questionnaires on child psychopathology and parental mental health as part of the routine diagnostic and outcome monitoring assessments. A parent or caregiver, usually the mother, received print copies of these questionnaires before the first appointment. Additionally, the parent questionnaire by Georg et al. (42) was used to record numerous demographic and therapy-relevant variables. It contains open and closed questions on areas such as birth complications, family situation, and reason for referral.

Parents self-reported the following reasons for referral: trouble sleeping through the night (86%), issues with child development or other (37%), defiant behavior, crying episodes or aggressive behavior (35%), frequent and persistent crying (34%), eating and feeding problems (28%), and anxiety, separation anxiety or clinging behavior (10%). A total of 22% of children had already received treatment elsewhere.

### 2.2 Questionnaire for Crying, Feeding and Sleeping (QCFS)

The Questionnaire for Crying, Feeding and Sleeping [QCFS, (43)] was used to assess early childhood psychopathology in infants aged 0–1.5 years. Parents rated 49 items on a 4-point Likert scale, ranging from “never or hardly ever” to “always or daily”, to determine the child's regulatory problems and three subscales (43). This study utilized the scales to measure various aspects of infant behavior and parental distress. The first scale, consisting of 24 items, measures crying, fussing, sleeping behavior, and parental perceptions (43, 44). An additional 13 items assess infant feeding problems, parental stress during feeding, and concerns about infant weight (43, 44). The third scale consists of 12 items that measure child-parent co-regulation and soothing attempts (43). The questionnaire has been validated for children aged 0–3 years (43). In this study, Cronbach's alpha ranges from .71 to .87 for the subscales and the overall scale. Pre-pandemic data was not available for 0- to 1.5-year-olds, as this questionnaire was only added to the routine data collection later.

### 2.3 Child Behavior Checklist 1½–5 (CBCL)

Clinical symptoms relevant for children aged 1.5 years and older include emotional reactivity, attention problems, aggressive behavior, anxiety, depressiveness, and social withdrawal (7). Furthermore, sleep problems can persist as part of psychopathological symptoms even in older children (7). Therefore, the Child Behavior Checklist 1½–5 (CBCL) was used to assess child psychopathology and behavioral problems in children aged 1.5–3 years (7, 45). Its 99 items have response options of “not applicable”, “somewhat or sometimes applicable”, and “accurate or frequently applicable” and assess children's emotional reactivity, anxiety and depressiveness, somatic complaints, social withdrawal, aggressive behavior, attention problems, and sleep problems (7). The CBCL total sum score in this study has a Cronbach's alpha of .94.

### 2.4 Patient Health Questionnaire

The German version of the Patient Health Questionnaire measures parents’ depressiveness with 9 items and their generalized anxiety with 7 items according to the DSM-IV classification system (46, 47). The response options, including “not at all”, “on some days”, “on more than half of the days”, and “almost every day” indicate symptom frequency (47). Cronbach's Alpha for the depressiveness and generalized anxiety scale are .80 and .87, respectively.

### 2.5 Stringency Index

As part of the Oxford COVID-19 Government Response Tracker, the Stringency Index described the severity of the restrictions imposed by governments worldwide in response to the COVID-19 pandemic (48). School closures, home office orders, workplace closures, event cancellations, contact restrictions, public transportation restrictions, curfews, domestic and international travel restrictions, and public information campaigns were included in the calculation of the index (48). Hale et al. (48) quantified the severity of these measures and reported daily values by country. The values for each day were extracted for Germany and assigned to the corresponding survey days.

In this study, the values of the Stringency Index ranged from 0 to 85.19. To test Hypotheses 2 and 3, the stringency index was used as a continuous measure of the severity of pandemic-related restrictions on the day of the survey. To test Hypothesis 1, the study period was divided into phases of lockdowns and reopening. Families who assessed their children's symptoms and their own well-being during the lockdown phase were compared with those who visited the Outpatient Department of Family Therapy during periods of less severe restrictions. The Stringency Index was subjected to a median split to differentiate between lockdown and reopening phases with the relaxation of measures. Table 1 offers an overview of the identified phases. If the value on the survey day exceeded the median of 63.43, it indicated more restrictive measures, categorizing those days as part of a lockdown phase. If the value was below 63.43, the measures were considered less restrictive. Each family was assigned to either the lockdown or the reopening phase based on the date of their data assessment in relation to their first contact with the Outpatient Department of Family Therapy. The median was reached between June 18, 2020, and July 6, 2020. This period is assigned to the reopening phase as the index values are lower both before and after this time frame. During these 19 days, six families were surveyed. Values were set to 0 before the first reported COVID-19 case in Germany on January 27, 2020, and before the recording of the index (48).

**Table 1:** Classification of the study period into lockdown and reopening phases.

Phase	End of phase	Number of surveyed families with children aged 0–1.5	Number of surveyed families with children aged 1.5–3
Pre-pandemic	Mar. 20, 2020	1	20
Lockdown	May 05, 2020	3	1
Reopening	Nov. 29, 2020	48	25
Lockdown	Aug. 02, 2021	71	25
Reopening	Nov. 14, 2021	28	13
Lockdown	Dec. 31, 2021	14	0

Note that the classification of phases is determined by a median split of the Stringency Index (48).

The identified phases can be linked to specific policy measures. The survey period began on September 19, 2019. The first lockdown was initiated with the recall of 65,000 international travelers (49) and the nationwide closure of non-systemically relevant facilities on March 21, 2020 (50). It ended on May 16, 2020, with initial re-openings (51) and the official end of the first wave of infections (52). New contact restrictions led to the second lockdown phase on November 28, 2020 (53), which ended on August 2, 2021, with re-openings for vaccinated and recovering individuals (54). The third lockdown phase started on November 14, 2021, and continued until the end of the survey period on December 31, 2021. During this period, regulations regarding the wearing of masks were in effect, and in some instances, access was restricted to vaccinated, recovered, or tested individuals (55).

### 2.6 Sample

The clinical sample consisted of families who were given age-appropriate questionnaires on their children's early childhood psychopathology. Parents who did not provide complete information on their child's age and gender or who completed less than 75% of the questionnaires were excluded, following the recommendations of Collins et al. and Schafer (56, 57). Informed consent for study participation was obtained from *N* = 249 families. Table 2 provides a detailed list of the demographic variables. On average, the children were 14 months old (SD = 10 months), with 51.41% being female. The majority of parents (74.95%) were of German origin, and 88.25% were married. Furthermore, 21.40% of mothers and 5.83% of fathers reported a history of mental disorders in their lifetime.

**Table 2:** Demographics of the final study sample.

	Children aged 0–1.5	Children aged 1.5–3
Sample size *N*	165	84
Child mean age in months (*SD*)	8.09 (4.41)	26.58 (6.66)
Number of girls (%)	83 (50.30)	45 (53.57)
Mother mean age in years (*SD*)	32.88 (4.37)	34.10 (4.65)
Father mean age in years (*SD*)	34.66 (5.54)	35.72 (6.35)
Number of mothers of German origin (%)	145 (88.96)	72 (86.75)
Number of fathers of German origin (%)	141 (88.13)	70 (88.61)
Number of families with more than one child (%)	54 (33.33)	41 (51.90)
Number of mothers with a university degree (%)	82 (50.93)	42 (52.50)
Number of fathers with a university degree (%)	77 (49.36)	35 (46.05)
Number of married mothers (%)	118 (71.95)	64 (78.05)
Number of married fathers (%)	116 (73.41)	64 (81.01)
Number of mothers with a history of mental disorder, lifetime (%)	35 (21.74)	17 (20.73)
Number of fathers with a history of mental disorder, lifetime (%)	8 (5.03)	6 (7.41)

### 2.7 Statistical analyses

All analyses were conducted using IBM SPSS Statistics version 27.0.1.0, G*Power version 3.1.9.7 (58) and R statistical software version 4.2.1 and its respective packages (59–66).

To address the issue of missing values in the questionnaires, a multiple imputation was performed based on a visual analysis indicating that the missing values were random (57, 67). The QCFS exhibited 3.92% of missing values, the CBCL 2.63%, and the Patient Health Questionnaire 0.87%.

To test Hypothesis 1, children of families who sought help during the lockdown and reopening phases were matched by age and gender using the propensity score (59, 68) to avoid bias. The group surveyed during the lockdown and the group surveyed during the reopening phases were compared using paired *t*-tests. Two phases were determined for group classification using the Stringency Index (48). The matching process resulted in 77 pairs of infants aged 0–1.5 years who were assessed by the QCFS, and 31 pairs of children aged 1.5–3 years assessed with the CBCL.

Given the likely gradual transitions between lockdown and reopening phases and the varying degrees of restrictions, the Stringency Index was utilized as a continuous measure in the subsequent analyses to capture the nuanced effects of pandemic restrictions. A regression analysis was used to investigate the impact of pandemic restrictions on early childhood psychopathology. Child age and gender were included as covariates to test Hypothesis 2, and parental mental health was added as a mediator to test Hypothesis 3. The proposed mediation model was first reported correlatively. To test the mediation model, a nonparametric bootstrap approach was performed with 10,000 Monte Carlo draws.

Finally, a hierarchical regression analysis was performed to identify the model with the optimal fit to explain early childhood psychopathology. The Stringency Index (48), the children's age and gender, and the parents’ generalized anxiety and depressiveness were included as possible predictors. The Akaike Information Criterion (69) was used as a metric for evaluating the inclusion of new predictors with a lower Akaike Information Criterion indicating a better model fit.

## 3 Results

Table 3 displays descriptive statistics for all questionnaires. The observed overall QCFS score was below the values from a clinical comparison sample [*M* = 2.23, *SD* = 0.35, (43)], as were the observed parental depressiveness [*M* = 11.7, *SD* = 5.00, (70)], and the parental generalized anxiety [*M* = 14.18, (71)]. In accordance with the established cutoff value of 64 proposed by (45), the mean sum score on the CBCL was below the threshold for clinical significance.

**Table 3:** Descriptive statistics of *n* = 165 children aged 0–1.5 and *n* = 84 children aged 1.5–3.

	*M (SD)*	Range
Sample with children aged 0–1.5 (*n* = 165)		
Overall QCFS score	1.38 (0.31)	0.45, 2.08
QCFS crying, fussing, sleeping	1.52 (0.42)	0.33, 2.54
QCFS feeding	.55 (0.51)	0.00, 2.46
QCFS co-regulation	2.02 (0.49)	0.58, 3.00
PHQ parental depressiveness	10.65 (5.11)	1, 25
PHQ parental generalized anxiety	8.53 (4.86)	0, 21
Sample with children aged 1.5–3 (*n* = 84)		
Total CBCL sum score	47.76 (23.90)	12, 132
PHQ parental depressiveness	7.91 (4.98)	0, 24
PHQ parental generalized anxiety	6.81 (5.00)	0, 18

QCFS, Questionnaire for Crying, Feeding and Sleeping (43); CBCL, Child Behavior Checklist 1½–5 (7); PHQ, Patient Health Questionnaire (47).

### 3.1 Early childhood psychopathology during lockdowns

Table 4 shows the results of Hypothesis 1. The score on Crying, Fussing and Sleeping Behavior on the QCFS (43) for infants aged 0–1.5 years was 0.08 points higher during lockdown than during reopening phases. The Co-regulation scale showed a mean difference of 0.09. In contrast, the mean score on the Feeding scale during lockdown was 0.06 points lower than the mean score during the reopening phase. A dependent samples *t*-test showed no significant differences for any of the subscales. Consequently, Hypotheses 1 a. (*1-β* = .47), Hypothesis 1 b. (*1-β* = .24), and 1 c. (*1-β* = .48) were rejected.

**Table 4:** Differences in early childhood psychopathology during phases of lockdown and phases of reopening.

	*M (SD)* during lockdowns	*M (SD)* during reopening	*t*	*p*	*d*	*CI*
QCFS crying, fussing, sleeping	1.57 (0.40)	1.49 (0.44)	1.12	.13	0.13	−0.04, Inf
QCFS feeding	0.52 (0.48)	0.58 (0.54)	−0.67	.51	0.08	−0.22, 0.11
QCFS co-regulation	2.08 (0.46)	1.99 (0.52)	1.12	.13	0.13	−0.04, Inf
CBCL sum score^a^	53.0 (28.3)	42.2 (19.5)	1.78	.04*	0.32	0.53, Inf

*df* = 76. QCFS, Questionnaire for Crying, Feeding and Sleeping (43); CBCL, Child Behavior Checklist 1½–5 (7).

^a^ *df* = 30.

* *p* < .05.

During lockdown, the CBCL sum score (7) for children aged 1.5–3 years was 10.83 points higher. A statistically significant increase in behavioral problems among toddlers was observed during the lockdown period compared to the reopening phases, as indicated by a paired *t*-test [*t* (30) = 1.78, *p* = .04, *d* = 0.32, 95% *CI* (0.53, Inf.), *1-β* = .67]. Therefore, Hypothesis 1 d cannot be rejected.

### 3.2 The impact of pandemic restrictions on early childhood psychopathology

**Figure 1:**
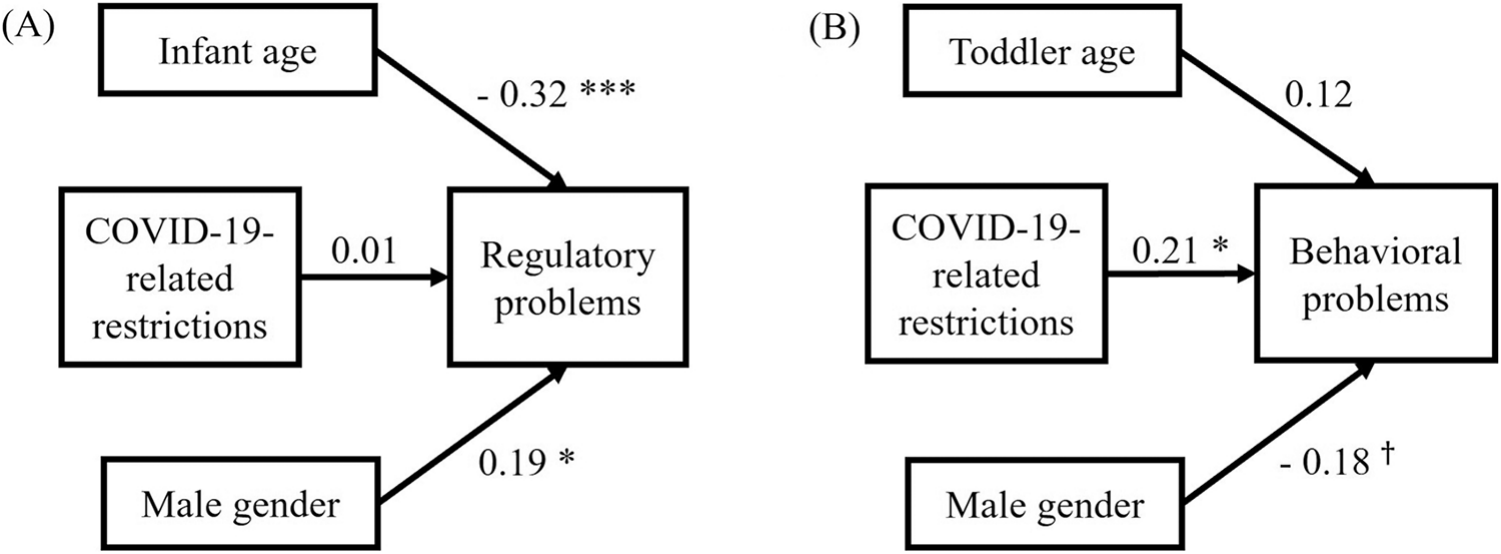
β-weights for children aged 0–1.5 years **(A)** and children aged 1.5–3 years **(B)** as postulated in hypothesis 2. Note that COVID-19-related restrictions were measured by the Stringency Index (48), infant regulatory problems by the Questionnaire for Crying, Feeding and Sleeping (43), and toddler behavioral problems by the Child Behavior Checklist 1½–5 (7). Model **(A)**: *R * = .13, *F*(3, 161) = 9.05, *p* < .001, Cohen's *f^2^* = 0.17, *1-β* = .997. Model **(B)**: *R^2^* = .08, *F*(3, 80) = 3.41, *p* = .02, Cohen's *f^2^* = 0.13, *1-β* = .78. ^†^*p* < .10. *p < .05. **p < .01. ***p < .001.

Figure 1 shows the relationship postulated in Hypothesis 2. In the infant sample between ages 0–1.5, the continuous Stringency Index measuring COVID-19 restrictions (48) was not a significant predictor of infant regulatory problems (*p* = .91). The covariates infant age (*p* < .001) and infant male gender (*p* = .01) significantly predicted infant regulatory problems, assessed with the QCFS. Therefore, Hypothesis 2 was rejected for infants between 0 and 1.5 years.

The same analysis for 1.5- to 3-year-olds showed a significant effect of the Stringency Index on child behavioral problems (*p* = .047), in accordance with Hypothesis 2 for this age group. Toddler age (*p* = .25) and gender (*p* = .09) did not influence the CBCL sum score.

### 3.3 The impact of parental mental health on early childhood psychopathology

**Figure 2:**
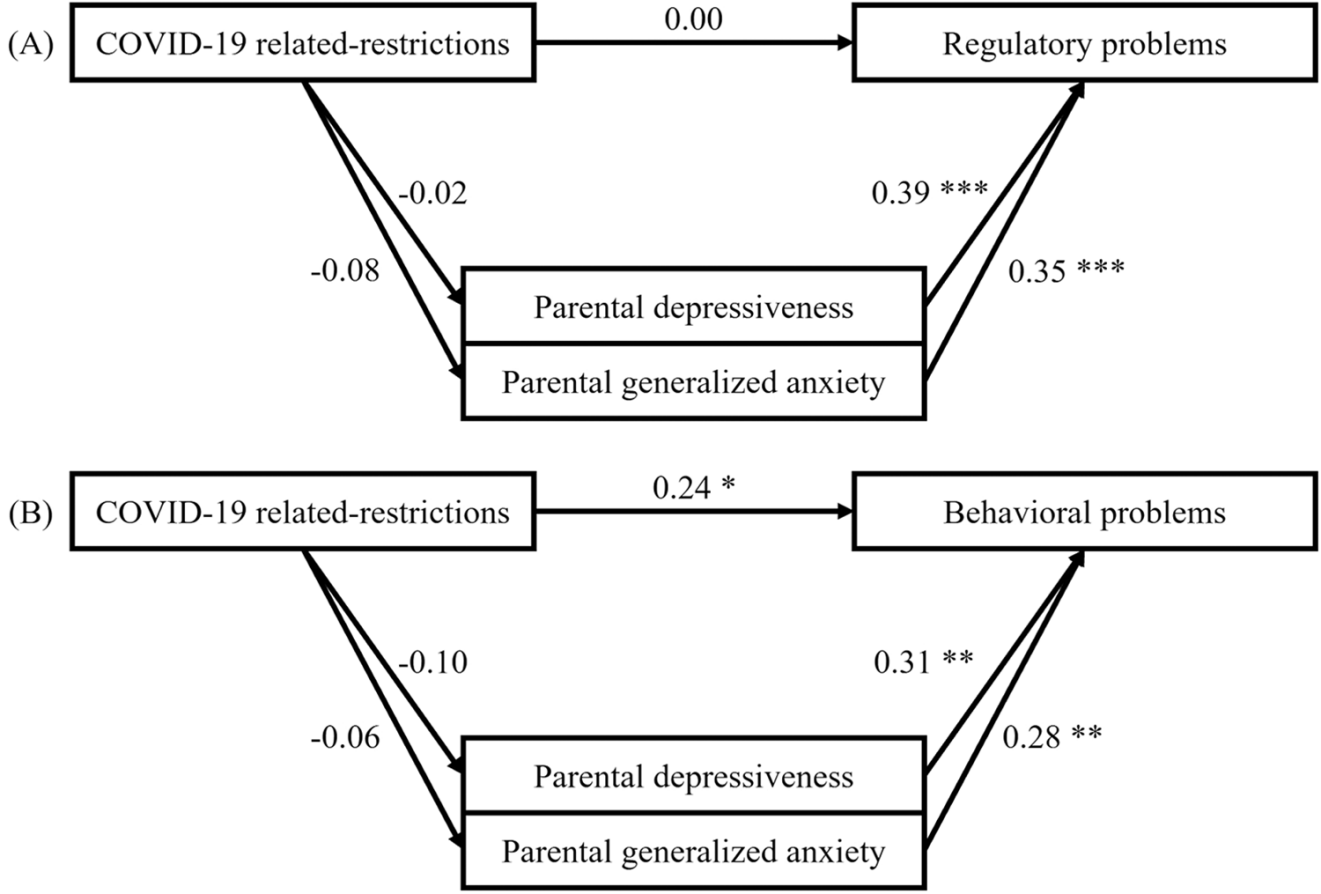
Correlations for *N* = 165 children aged 0–1.5 years **(A)** and *N* = 84 children aged 1.5–3 years **(B)** as postulated in hypothesis 3. Note that COVID-19- related restrictions were measured by the Stringency Index (48), infant regulatory problems by the Questionnaire for Crying, Feeding and Sleeping (43), toddler behavioral problems by the Child Behavior Checklist 1½–5 (7), and parental mental health by the Patient Health Questionnaire (47).

Figure 2 depicts the postulated relationships in Hypothesis 3 and the results of the mediation analyses with parental mental health as a mediator. The mean parental depressiveness as measured by the Parent Health Questionnaire (47) was 10.13 (*SD* = 5.47) during phases of lockdown and 9.36 (*SD* = 4.98) during phases of reopening. Similarly, the mean parental generalized anxiety was higher during lockdown (*M* = 8.04, *SD* = 4.97) compared to phases of reopening (*M* = 7.87, *SD* = 4.99). Pandemic restrictions did not correlate significantly with parental depressiveness (*r* (247) = .03, *p* = .67) nor with parental generalized anxiety (*r*(247) = −.004, *p* = .95).

For 0–1.5-year-old infants, the mediation analysis did neither reveal a main effect of the severity of pandemic restrictions on regulatory problems nor an indirect effect mediated by parental mental health [average direct effect of restrictions with depressiveness as a mediator: *β* = .01, *p* = .89, *CI* (−.12, .14); average causal mediation effect of depressiveness: *β* = −.01, *p* = .84, *CI* (−.06, .05); average direct effect of restrictions with generalized anxiety as a mediator: *β* = .03, *p* =68, *CI* (−.10, .16); average causal mediation effect of generalized anxiety: *β* = −.03, *p* = .37, *CI* (−.08, .03)]. Similarly, no mediation effect was observed for children aged 1.5–3 years. However, a main effect was identified for this age group, indicating that the severity of pandemic restrictions significantly predicted child behavioral problems [average direct effect of restrictions with depressiveness as a mediator: *β* = .27, *p* = .01, *CI* (.08, .46); average causal mediation effect of depressiveness: *β* = −.03, *p* = .37, *CI* (−.11, .46); average direct effect of restrictions with generalized anxiety as a mediator: *β* = .26, *p* = .01, *CI* (.07, 0.44); average causal mediation effect of generalized anxiety: *β* = −.02, *p* = .63, *CI* (−.09, .05)]. Nevertheless, Hypothesis 3 was rejected for both age groups.

### 3.4 Best-fit model for explaining early childhood psychopathology

Table 5 shows the best-fitting model for predicting early childhood psychopathology. The first hierarchical regression analysis indicated that infant age and gender, as well as parental depressiveness, predicted regulatory problems in 0- to 1.5-year-old infants. On average, for each additional month of age, the score on the QCFS was lower by 0.06 standard deviations, indicating fewer regulatory symptoms in older infants. Parents of male infants reported higher scores by 0.35 standard deviations. An increase in parental depression of one standard deviation was associated with a 0.37 standard deviation increase in the QCFS score. Overall, the model (*p* < .001) explained 26.35% of the variance, as measured by the adjusted *R^2^*.

**Table 5:** Results of a hierarchical regression analysis to explain early childhood psychopathology.

	*β*	*p*
Sample with children aged 0–1.5		
PHQ parental depressiveness	0.37	<.001***
Age in months	−0.29	<.001***
Gender (male)	0.18	.008**
Total model^a^	–	<.001***
Sample with children aged 1.5–3		
PHQ parental depressiveness	0.32	.002**
Stringency index	0.26	.013*
Gender (male)	−0.17	.09^†^
Total model^b^	–	<.001***

Note that COVID-19-related restrictions were measured by the stringency index (48), infant regulatory problems by the questionnaire for crying, feeding and sleeping (43), toddler behavioral problems by the child behavior checklist 1½–5 (7), and parental depressiveness by the patient health questionnaire [PHQ, (47)]. All measures were *z* standardized.

^a^ *R^2^* = .26, *F*(3, 161) = 20.55, *1-β* = .99.

^b^ *R^2^* = .17, *F*(3, 80) = 6.55, *1-β* = .98.

^†^ *p* < .10.

* *p* < .05.

** *p* < .01.

*** *p* < .001.

The severity of restrictions imposed during the pandemic, parental depressiveness, and children's age and gender significantly predicted the prevalence of behavioral problems in 1.5- to 3-year-olds. The severity of behavioral symptoms increased by 0.02 standard deviations for each additional month of age and decreased by 0.34 standard deviations for male toddlers. Furthermore, when pandemic-related limitations or parental depressiveness were higher by one standard deviation, symptoms were more severe by 0.26 and 0.32 standard deviations, respectively. The adjusted *R*^2^ indicated that the model explained 16.70% of the variance.

## 4 Discussion

The negative effects of the COVID-19 pandemic on the mental health of families and young children have been demonstrated empirically several times. Less is known about the effects in clinical groups. The present study aimed to investigate the impact of the COVID-19 pandemic and parental mental health on early childhood psychopathology in a clinical sample seeking parent-infant/toddler psychotherapy due to child behavior problems. Consistent with Bronfenbrenner's (15) theoretical framework of protective and risk factors for child development, this study postulated that the changes in care and for families due to the pandemic negatively affected early childhood psychopathology. This effect was expected to be partially mediated by parental mental health. Our results showed that pandemic restrictions and lockdowns negatively affected young children aged 1.5–3 years, whereas an effect on children aged 0–1.5 years was not found. The symptoms of early childhood psychopathology became more pronounced in the older age group under more severe restrictions on daily life. Contrary to expectations, this effect was not mediated by parental generalized anxiety and depressiveness. Nevertheless, parental depressiveness negatively correlated with early childhood psychopathology in all age groups. More severe restrictions due to the pandemic, child female gender, and higher levels of parental depressiveness were predictive of increased behavioral problems in children aged 1.5–3 years, including emotional reactivity, anxiety, depressiveness, somatic complaints, social withdrawal, aggressive behavior, attention problems, and sleep problems. Younger age, child male gender, and high parental depressiveness, but not pandemic restrictions, predicted regulatory problems in children aged 0–1.5 years.

The findings regarding the relation of child age and gender and regulatory problems in infants between the ages of 0 and 1.5 years align with those of previous literature. Older and female infants showed fewer symptoms of regulatory problems. Similarly, previous studies independent of the pandemic reported lower prevalence rates for older and female infants (8, 10–14). Conversely, in the literature, behavioral problems in 1.5- to 3-year-old toddlers, particularly aggressiveness and attention problems, are more frequently observed in male infants (7, 9). However, the findings of this study indicated that female gender was a marginally significant predictor of behavioral problems in 1.5- to 3-year-old toddlers. This discrepancy may be because the present study utilized the CBCL sum score without examining specific behavioral problems. In future studies on the impact of pandemics, early childhood psychopathology in toddlers should be considered in a more differentiated way, for example in externalizing and internalizing problems, to investigate possible gender-specific correlations.

Despite overwhelming evidence from other studies (31, 32), parental mental health did not significantly correlate with the severity of the pandemic's restrictions. One reason for this may be that parents who stayed home to care for their children did not have to commute to work, or who worked from home may have experienced less daily stress despite the pandemic. However, as expected, parental depressiveness significantly predicted early childhood psychopathology at all ages. Consequently, this study contributes to the existing research that showed a robust correlation between parental mental health and early childhood psychopathology (8, 24, 28). This finding also aligns with Bronfenbrenner's framework, which suggests that the family microsystem significantly influences child mental health and development (15). According to our results, this effect is not linked to pandemic restrictions. For children aged 1.5–3 years, parental depressiveness and the severity of COVID-19 restrictions were independent predictors of early childhood psychopathology.

In children between the ages of 1.5 and 3, this study found that the impact of the COVID-19 restrictions on the extent of early childhood psychopathology was as expected. During periods of increased restrictions on routines and changes in daily life children in this age range exhibited more severe behavioral problems. The relation could likely result from fewer opportunities for physical activity, reduced peer contact, and more frequent changes in childcare arrangements. First, literature suggests the important role of physical activity for the mental health of children during the pandemic. Two meta-analyses confirm that physical activity can be a protective factor for behavioral problems and child mental health during the pandemic in school-age children (72, 73). Despite the effect of the pandemic on physical activity, it is plausible that the level of physical activity declined, particularly during the winter months when restrictions were typically more stringent. Therefore, the impact of reduced physical activity may extend beyond the restrictions imposed by the pandemic. Second, reduced contact with peers during early childhood may coincide with increased social withdrawal and depressiveness (74), hindering the development of skills such as managing one's aggressive behavior, emotional reactivity, and attention regulation (75–77). Third, families may have experienced many changes in childcare responsibilities during the pandemic. As a result, children had to adapt more frequently which could have contributed to more child behavioral problems. As childcare external to the family is more prevalent among older children in Germany (78), changes in childcare, in addition to reduced physical activity and peer contact, could have particularly affected 1.5- to 3-year-olds.

This study found no evidence to support the prediction that regulatory problems in 0– to 1.5-year-olds would increase along with pandemic restrictions and lockdowns in a clinical sample. Gadermann et al. (36) found that 22% of parents reported more family conflicts, while nearly 50% experienced increased feelings of closeness within the family. It is possible that many families experienced positive consequences of the pandemic restrictions, which may have been more pronounced for children under 1.5 years of age when families care for their children at home more often. In the United States, 65% of parents working from home reported having childcare responsibilities while working (79). Due to isolation, families may have experienced a closer bond that may have strengthened parental interactional and co-regulatory skills, which may explain why regulatory problems in infants aged 0–1.5 years did not worsen during high pandemic restrictions. Furthermore, pre-pandemic data was not available for 0- to 1.5-year-olds. The pandemic may have had an overall detrimental effect on infants’ regulatory behaviors that could not be detected with the available data. However, for children aged 1.5–3 years, 21% of data was collected before the first case of COVID-19 was reported in Germany and before the onset of the first lockdown. The reported effects of pandemic restrictions on behavioral problems may have been dominated by large differences between pre-pandemic data and data collected during the pandemic.

Overall, parents reported more infant regulatory problems for newborns with symptoms decreasing with each month of age. Thus, newborns appear particularly vulnerable to regulatory difficulties, just as older toddlers are more vulnerable to behavioral problems. This finding allows institutions to provide targeted interventions to young families.

Most importantly, this study highlights the significance of family support services, particularly during times of crisis. The negative effects of lockdowns on child behavioral problems could add to the burden on young families. Mental health services are inadequate in many regions, even during times of non-crisis (80). Parent-infant/toddler interventions can help parents cope with the new challenges posed by children with regulatory disorders (8). Similarly, early prevention efforts in times of crisis should not only target parents of infants under 1 year of age but also parents with older children. It is possible, that toddlers experience the negative effects of lockdowns more directly through changes in their familiar routines, physical activity and peer interactions. Finally, parental mental health support should be expanded since parental depressiveness has been shown to be an important predictor of early childhood psychopathology.

### 4.1 Limitations and future research

Although this study was able to include parental mental health as a mediator, future research should consider other factors that contribute to the development and maintenance of early childhood psychopathology during crisis, given that the explained variance of the included factors was less than 27%. For example, future research should include measures of physical activity, peer contact, and changes in child and family routines. Furthermore, 40% of the families in this study reported having more than one child. Thus, it is difficult to attribute poor parental mental health solely to the child presented to the Outpatient Department of Family Therapy. Research suggests that children with more older siblings have fewer regulatory problems (19). Moreover, factors such as loneliness and parental stress have been associated with poor parental mental health (3, 31), which in turn may negatively affect early childhood psychopathology. Changes in routines, child peer contact, number of children, parental loneliness, and stress were not included in this analysis. Considering these factors may contribute to a better understanding of the adverse effects of the pandemic on early childhood psychopathology and should be included in future research. The inclusion of such variables may also help explain the different findings for 0- to 1.5- and 1.5- to 3-year-olds. Nevertheless, this study addresses an important gap in the literature by investigating early childhood psychopathology in the context of the pandemic while considering parental mental health as an essential predictor of child mental health.

This study's design did not allow for a causal interpretation. For instance, it is not possible to conclude that worse parental mental health has a negative influence on early childhood psychopathology. Negative effects from early childhood psychopathology on parental mental health (81) and even reciprocal effects are possible (82). The hypotheses of this study, however, were formulated based on Bronfenbrenner's framework, which assumes that parental mental health, as part of the child's microsystem, influences child wellbeing (15).

Furthermore, a more diverse sample should be included in future research. Parents with low levels of education and migrant backgrounds were particularly affected by the pandemic (1, 39). These characteristics were not included as predictors in this study due to their limited variability in this sample. Most families reside in the Heidelberg region, have an above-average socio-economic status, and are of German origin. This may lead to confounding results in the study, as the impact of the pandemic disproportionately affected racial and ethnic minorities (39). However, since the sample is from the same region, restrictions were the same for all families. Some COVID-19 policies were partly incidence- and state-dependent [e.g., (83)]. Nevertheless, future research should aim to obtain a more diverse sample and investigate the reported relationships with differing socioeconomic variables.

Additionally, it is important to note that the participants in this study were drawn from a clinical sample. Consequently, the prevalence of parental and child psychopathology is likely higher than that in the general population, and the effects and relationships observed in this study may differ accordingly. It is important to note, however, that compared to other samples children in our study had lower levels of psychopathology. The sample mean scores on all scales of the QCFS for children aged 0–1.5 years were lower than those of the healthy and clinical comparison samples (43). Furthermore, three-quarters of children aged 1.5–3 years were reported to have behavioral problems below clinically relevant levels (45). Nevertheless, this study addresses a gap in the literature by examining a clinical sample that differs from the samples used in most other studies conducted during the COVID-19 pandemic (3).

Although this study surveyed parents with young children over several months, the cross-sectional study design has several limitations. This study was unable to specifically examine intraindividual differences. Furthermore, pre-pandemic data for this study was available for 1.5- to 3-year-old toddlers, but not for younger infants. Considering pre-pandemic data is essential for assessing the overall impact of the pandemic. To minimize the potential effects of confounding variables on the results, this study matched children regarding age and gender. Nonetheless, future research should conduct longitudinal studies and explore trajectories throughout early childhood.

Another limitation of this study design is that it only allowed us to identify an increase in early childhood psychopathology and parental mental health issues across the pandemic. The pandemic may have imposed a greater burden on the general population, resulting in an increased frequency of cases. Furthermore, families that would not otherwise be affected by early childhood psychopathology or parental mental health issues may have experienced a burden due to the pandemic and thus sought psychological help. Future studies should investigate whether more families seek help in times of crisis, such as the COVID-19 pandemic. However, this will depend on the availability of and barriers to mental health services. Additionally, it would be of interest to determine whether there is a higher prevalence of early childhood and parental psychopathology in the general population during periods of pandemic.

Furthermore, the Stringency Index may not be the most appropriate measure to assess the pandemic's impact on psychopathology. The division of the time periods during the pandemic into phases of lockdowns and reopening was based on the implementation of policy measures and a median split. First, the personal burden imposed by the pandemic may have accumulated over time, rather than being directly proportional to the severity of restrictions. Mental disorders often have incubation periods (84), and the negative effects of quarantine can persist longer than the quarantine itself (85). Consequently, the Stringency Index, which is used to measure the severity of restrictions, may not always be an appropriate metric for identifying the consequences of the COVID-19 pandemic. This is because the effects of the pandemic are often not immediately apparent, especially given the complex causes underlying the development of mental health issues and early childhood psychopathology. Second, restrictions imposed at the onset of a lockdown may have appeared to cause more significant disruptions to personal life than the same measures imposed at the end of a lockdown. Habituation effects could have reduced the perceived threat of the prolonged second COVID-19 wave. Therefore, personal restrictions and perceptions may provide more informative insights than policy measures. For example, a case of COVID-19 in the family or pre-existing medical conditions could have influenced the perceived threat of the pandemic, while the subjective feeling of isolation could have altered the perceived severity of the measures. Future research should include subjective measures of the perceived severity of the COVID-19 measures. Nevertheless, the Stringency Index remains the most objective measure of the severity of COVID-19 restrictions for each day.

Both parental mental health and the extent of early childhood psychopathology were assessed through parent reports which are not always accurate and objective (86). Some parents may have responded in a socially desirable manner, reporting fewer family problems than exist. This is supported by the fact that the sample mean scores on all scales of the QCFS were lower than those of the healthy and clinical comparison samples (43). This finding is particularly surprising given that the families were experiencing such high levels of distress that they were open and motivated to seek treatment at the Outpatient Department of Family Therapy. Further research is necessary to confirm the findings of this study, particularly with clinically diagnosed disorders in both children and parents using standardized clinical assessments, like the DC: 0–5 (87).

In general, the power was above .95 for most of the analyses. However, the power for the dependent sample *t*-test comparing the lockdown and reopening phases was between .24 and .67. These low power levels (88) may account for the absence of any observed effects of lockdowns on regulatory disorders in children aged 0–1.5 years. In addition, the multiple regression analysis explaining behavioral problems in children aged 1.5–3 years with toddler age and gender as covariates had a power of 0.78. Notwithstanding the lower power, parental depressiveness, the level of restrictions during the pandemic, and the children's gender all significantly predicted behavioral problems.

## 5 Conclusion

The study found no increase in infant psychopathology (0–1.5 years of age) or in parental depressiveness and generalized anxiety in a clinical sample during the pandemic overall. However, older children (1.5–3 years of age) showed more behavioral problems during more severe pandemic phases and with increasing severity of pandemic restrictions. Furthermore, early childhood psychopathology was strongly associated with parental depressiveness and anxiety, independent of the pandemic. Further longitudinal research is needed to fully understand the impact of the pandemic on infants and toddlers and its interaction with parental mental health. The significant effects of covariates like child age and gender on early childhood psychopathology, suggest that more developmental, environmental and contextual variables, such as care arrangements, children's peer contacts, and parents’ occupational solutions, should be included in analyses to improve the prediction of early childhood psychopathology during the pandemic. The present study highlights the importance of implementing parent-child interventions in early childhood, beyond infancy, to support young families in times of crisis. Because of the robust association between parental mental health and early childhood psychopathology, parent-child support services focused on parents should be available for all ages during times of crisis and beyond.

## Data Availability

The datasets presented in this article are not readily available because the personal data of surveyed families is not published with the article. Requests to access the datasets should be directed to Anna Georg, anna.georg@med.uni-heidelberg.de.
